# Effective Defense of Aleppo Pine Against the Giant Scale *Marchalina hellenica* Through Ecophysiological and Metabolic Changes

**DOI:** 10.3389/fpls.2020.581693

**Published:** 2020-12-10

**Authors:** Mariangela N. Fotelli, Fani G. Lyrou, Dimitrios N. Avtzis, Daniel Maurer, Heinz Rennenberg, Gavriil Spyroglou, Andrea Polle, Kalliopi Radoglou

**Affiliations:** ^1^Forest Research Institute, Hellenic Agricultural Organization Demeter, Thessaloniki, Greece; ^2^Chair of Tree Physiology, Institute of Forest Sciences, University of Freiburg, Freiburg im Breisgau, Germany; ^3^Department of Forest Botany and Tree Physiology, Georg-August University of Göttingen, Göttingen, Germany; ^4^Department of Forestry and Management of the Environment and Natural Resources, Democritus University of Thrace, Orestiada, Greece

**Keywords:** Aleppo pine, infestation, *Marchalina hellenic*a, gas exchange, total C and N, δ^13^C, sugars, metabolic changes

## Abstract

Aleppo pine (*Pinus halepensis*) is widely distributed in the Mediterranean region and in other areas of the world, where it has been introduced due to its adaptive capacity to xerothermic conditions. The giant pine scale *Marchalina hellenica* often infests Aleppo pine, as well as other pines, in several southeastern European countries, causing pine declines. When combined with the expected intensified heat and drought events in eastern Mediterranean, the impact of this biotic parameter on the host pines may be exacerbated. The importance of understanding the defense mechanisms of Aleppo pine is emphasized by the recent invasion of the pine scale in new regions, like Australia, lacking the insect’s natural enemies, where more intense negative effects on pine species may occur. To date, Aleppo pine’s physiological responses to the infestation by *M. hellenica* are largely unknown. This study aimed at assessing the responses of Aleppo pine to the giant pine scale attack, both on an ecophysiological and a metabolic level. For this purpose, gas exchange, needle water status, and carbon and nitrogen content were measured during 1 year on healthy and infested adult trees. M etabolic profiling of Aleppo pine needles was also performed before, during, and after the high feeding activity of the insect. The maintenance of stable relative water content, δ^13^C signatures, and chlorophyll fluorescence in the needles of infested pines indicated that infestation did not induce drought stress to the host pines. At the peak of infestation, stomatal closure and a pronounced reduction in assimilation were observed and were associated with the accumulation of sugars in the needles, probably due to impaired phloem loading. At the end of the infestation period, tricarboxylic acids were induced and phenolic compounds were enhanced in the needles of infested pines. These metabolic responses, together with the recovery of photosynthesis after the end of *M. hellenica* intense feeding, indicate that in the studied region and under the current climate, Aleppo pine is resilient to the infestation by the giant pine scale. Future research should assess whether these promising defense mechanisms are also employed by other host pines, particularly in regions of the world recently invaded by the giant pine scale, as well as under more xerothermic regimes.

## Introduction

Aleppo pine (*Pinus halepensis* Mill.) is a widespread species that is native to the Mediterranean basin (Euro+Med, 2006) but has also become naturalized in southern Australia, southern Africa, New Zealand, and southwestern United States (Queensland Government, 2016). It is a forest tree of economic importance in many Mediterranean countries, used for the production of wood, resin, health, and nutrition products (Chambel et al., 2013; Salim et al., 2019).

The wide distribution of Aleppo pine to warm and dry environments is supported by the species’ isohydric responses, bimodal active growth, and high regeneration potential ensuring its survival and adaptation under diverse abiotic stresses, including drought, heat, and wild fires (Klein et al., 2011; Calvo et al., 2013; Choury et al., 2017; Gazol et al., 2017; Fotelli et al., 2019). Previous studies showed that Aleppo pine is able to seasonally adjust its photosynthetic activity to limit water losses and take advantage of even short-term favorable environmental conditions (Sperlich et al., 2015; Choury et al., 2017; Fotelli et al., 2019). However, intensified and more frequent heat and drought events are expected in the Mediterranean area (García-Ruiz et al., 2011; Hoegh–Guldberg et al., 2018) and are linked to growth declines, particularly at its eastern part (Sarris et al., 2007). Pines may survive drought by adopting wide hydraulic safety margins, which can be at the expense of growth but are negatively affected by temperature increases on the long term (Carnicer et al., 2013).

Such xerothermic conditions are known to predispose pines to insect attacks (Gaylord et al., 2013). Thus, the biotic stress caused by the giant scale *Marchalina hellenica* Gennadius (Hemiptera: Marchalinidae) may be a key parameter affecting the performance of Aleppo pine, especially in the light of climate change. *M. hellenica* is a sap-sucking insect that feeds on adult trees of several pine species (mostly *P. halepensis* and *Pinus brutia* Ten and to a lesser extent *Pinus sylvestris* L., *Pinus nigra* Arnold, and *Pinus pinea* L.), but also on fir (*Abies cephalonica* L.) (Bacandritsos et al., 2004; Yeşil et al., 2005; Gounari, 2006; Tsagkarakis and Emmanouel, 2016). The insect is native to south-eastern Europe but has recently invaded Croatia and southern Australia where it feeds on *P. halepensis*, *P. pinea*, and *Pinus radiata* D. Don (Eppo Global Database, 2015; Masten Milek et al., 2019; Avtzis et al., 2020).

In Greece and Turkey, *M. hellenica* is highly valued in apiculture since honey bees are feeding on its honeydew to produce most of the world’s pine honey (Gounari, 2006; Bouga et al., 2011; Ünal et al., 2017). Especially in Greece, c. 60–65% of the country’s pine honey is produced by the scale insect’s secretions (Thrasyvoulou and Manikis, 1995). Due to its economic importance, the insect has been deliberately introduced in new regions in Greece in the late 90s, as well as in the Italian island of Ischia (Eppo Global Database, 2006; Mendel et al., 2016). This practice resulted in the distribution of *M. hellenica* in both low altitude and mountainous regions, as well as in several islands in Greece (Bacandritsos et al., 2004; Avtzis et al., 2020). The high population density of the giant pine scale was associated with adverse effects on the host pines, such as growth decline, branch and foliage desiccation, and, in combination with other abiotic and biotic secondary stresses, tree mortality (Gallis, 2007; Mendel et al., 2016). A negative impact on growth of *P. brutia* has also been documented in Turkey (Yeşil et al., 2005), where no additional artificial infestation with *M. hellenica* was implemented.

Similar or greater impacts on infested Aleppo pines and other host conifers are most probable in regions lacking the insect’s natural enemies. In Australia, the economic and ecological impacts of infestation by *M. hellenica* on *P. radiata*, comprising c. 74.5% of the country’s softwood plantations area, are still unquantified (Avtzis et al., 2020). To counteract the problem, biological control of the insect has been lately initiated in Australia, since destruction of infested trees and application of chemical agents were insufficient to control the pest (Avtzis et al., 2020). In Italy, phytosanitary measures are taken to prohibit the expansion *M. hellenica* beyond the island of Ischia, while in Greece, control means are sporadic, mainly in urban areas, due to the insect’s importance in apiculture (Eppo Global Database, 2006).

Therefore, assessing the physiological consequences for Aleppo pine when infested by *M. hellenica* is critical to explore the responses of this forest tree to long-term interaction with the giant pine scale in a changing climate. Still, current knowledge about these consequences is limited. The only reported ecophysiological effect of infestation is reduced stomatal conductance in Aleppo pine needles, measured at a single time point (Petrakis et al., 2010). Furthermore, infestation of *P. halepensis* by another sap-sucking pine scale (*Matsucoccus josephi*) has been reported to cause desiccation, attributed to tracheid injury and blocked water transport (Mendel and Liphschitz, 1988). In addition to ecophysiological approaches, metabolic profiling is essential to understand the mechanisms mediating physiological changes in Aleppo pine upon attack by *M. hellenica*. Plant–insect interactions are known to induce the biosynthesis of defense compounds depending on central metabolic pathways for energy demands; thus, diverse changes in both primary and secondary plant metabolism can be expected (e.g., Tian et al., 2012; Nishida, 2014; López–Goldar et al., 2018; Kortbeek et al., 2019; Papazian et al., 2019). However, to date, metabolite abundances and their changes upon infestation by *M. hellenica* were studied only for Aleppo pine resin terpenoides (Roussis et al., 2001; Mita et al., 2002).

In the present study, we analyzed a range of physiological parameters to assess the gas exchange patterns and water balance of healthy and infested *P. halepensis* trees during an entire year. Moreover, during the peak of the giant scale’s activity, we performed metabolic profiling in the host tree needles to determine the consequences of infestation by *M. hellenica* on the abundance of major groups of metabolites and to gain an understanding of metabolic reactions that potentially could counteract the giant scale infestation. Thus, we tested the hypotheses that long-term infestation of Aleppo pine by the pine giant scale leads (a) to impaired needle gas exchange and water status and (b) to metabolic disorders in response to this biotic stress.

## Materials and Methods

### Study Areas

In northern Greece, Aleppo pine is naturally distributed solely at the region of Halkidiki (Fady et al., 2003; De Luis et al., 2013). Thus, we selected a c. 70-year-old Aleppo pine forest stand infested by *M. hellenica* in Sane, Halkidiki, Greece (40°06.21′ N, 23°18.79′ E, 15 m.a.s.l., zero slope) as the infested study site. According to macroscopic observations before the initiation of the study, most pines at this site were to some extent infested by the giant pine scale and it could not be ensured that the few unaffected individuals would not get infested during the study period. In addition, field surveys at neighboring low-elevation Aleppo pine stands with similar growth conditions and biometric traits revealed that infestations by the insect were extended and completely unaffected forest stands could not be identified. For this reason, a 58-year-old Aleppo pine plantation in the experimental station of the Forest Research Institute (FRI), Thessaloniki, Greece (40°30.57′ N, 23°04.79′ E, 30 m.a.s.l., zero slope) comprising healthy trees was used as the control site of the study. The height and diameter at breast height (DBH) of the studied sites are presented in Table 1. Both pine sites are established at low elevation (15–30 m.a.s.l) and zero slope and share similar climatic and soil conditions (Table 1).

**TABLE 1:** Biometrical, climatic^1^, and soil traits of the two Aleppo pine study sites.

	*FRI – control*	*Sani – infested*
Mean tree height ± SE [m]	24.8 ± 0.4	16.1 ± 0.6
Mean DBH ± SE [cm]	38.9 ± 1.0	44.9 ± 3.3
Mean air temperature [°C]	15.5	16.0
Annual rainfall [mm]	565.6	559.8
Aridity index^2^	0.88	0.84
Soil texture^3^ [0–50 cm depth]	SCL–CL	SL–SCL
Soil pH [0–50 cm depth]	7.6–7.7	7.5–7.9
Soil type^4^	Vcc	Vcc–Lc

*^1^The climatic parameters presented (air temperature, rainfall, and aridity index) are mean values of the periods 2002–2018 and 2008–2018 for the control and the infested site, respectively. ^2^Mean annual aridity index was calculated as the ratio of potential evapotranspiration (PET) to precipitation (rainfall). PET was calculated according to Allen et al. (1998). ^3^SCL, sandy clay loam; CL, clay loam; SL, loamy sand. ^4^Vcc, Calcaric chromic vertisols; Lc, Chromic luvisols. Soil type was defined according to European Soil Bureau Network (2005).*

To characterize the growth conditions during the study period, air temperature (RHT2nl, Delta–T Devices Ltd., Cambridge, United Kingdom) and precipitation (AR100 and RGB1, EM United Kingdom) were recorded on an hourly basis and data logged by automated meteorological stations were established close to the study sites.

### Sampling Campaigns and Physiological Measurements

Within an area of c. 0.5 ha at each site, six and seven dominant, non-neighboring Aleppo pine trees were used at the control and infested site, respectively. Selection of the infested trees in the field depended on the macroscopic evaluation of *M. hellenica* attack as indicated by the cotton-like wax secreted by the scale that was found on the tree branches. Measurements and sampling were conducted at both study areas for 1 year (August 2018–July 2019) on a monthly basis, on two consecutive sunny days. If cloudy conditions occurred on the second measuring day (October 2018, January 2019, and April 2019), measurements and sampling took place on the next sunny day.

Two fully sunlit branches were collected from the upper crown of each tree using a telescopic scissor. Immediately after cutting, each branch was placed in a water container and was re-cut in water according to the methodology applied for assessing chlorophyll fluorescence (Pollastrini et al., 2016) and gas exchange (Bahar et al., 2017; Fyllas et al., 2017, 2020) of adult trees. While each cut branch was submerged in water, maximum photosynthesis (*A*_*max*_) and stomatal conductance (*g*_*s*_) were measured on 1-year-old needles using the Li6400XT portable gas exchange analyzer coupled to a Li6400–40 fluorescence chamber (LI-COR, NE, United States). Fully expanded needles of each cut branch were placed in the 2-cm^2^ cuvette, and the cuvette was firmly closed to ensure stable measurements. When no firm sealing of the cuvette is achieved, Blu-tack is frequently applied (e.g., Sperlich et al., 2015) but was not required in the present study, as no CO_2_ response curves were conducted. The needles were carefully arranged so that no overlapping of needles occurred and the cuvette area was fully covered. For the measurements, PAR was set at 1000 μmol m^–2^ s^–1^, based on light response curves conducted in spring, which showed that maximum photosynthesis was achieved at this PAR level (data not shown). Although optimal PAR may vary seasonally, we chose to keep the same PAR level throughout the study for comparability reasons. CO_2_ flow rate in the chamber was set to 300 μmol s^–1^ and reference CO_2_ at 400 ppm. Depending on the seasonal fluctuation of ambient air temperature, chamber temperature was maintained at a range of 17–25°C. Sperlich et al. (2019) showed that net assimilation of *P. halepensis* was acclimated to the seasonal changes in growth temperature, as long as no heat waves occurred. We generally avoided hot summer days and initiated summer measurements the latest till 10:00 a.m. Likewise, very cold winter days were avoided and measurements in winter days were initiated later. In general, measurements were performed between 10:00 and 12:00 in summer and spring and between 11:00 and 13:00 in winter and autumn days. During each measurement, *A*_*max*_ and *g*_*s*_ values were monitored and, when stable, 5–8 recordings were logged at c. 3- to 5-s intervals, and used to calculate mean values. On average, stable values of gas exchange parameters were achieved within 10–20 min after the needles were put in the cuvette. Intrinsic Water Use Efficiency (WUE_*i*_), was calculated by the ratio of *A*_*max*_ to *g*_*s*_. A pre-test performed with sunlit low canopy branches of three Aleppo pines grown at the control site (FRI) showed that *A*_*max*_ remained stable half an hour after abscission (Supplementary Figure 2). *A*_*max*_ was measured, using the settings described above, before branch abscission, as well as 10 min, 20 min, and 30 min after abscission.

After completion of gas exchange measurements, a subset of needles from each branch was collected for the determination of relative water content (RWC;%). RWC was calculated according to the formula of Weatherley (1950):

(1) RWC=(FW-DW/TW-DW)×100

where FW is the needles’ fresh weight, DW is the needles’ dry weight after oven-drying (75°C, 5 days), and TW is the turgor weight after the needles were saturated in water (5°C, 24 h).

For chlorophyll fluorescence measurements, the branches were placed in black bags for dark adaptation, transferred in cool boxes, and stored in a cold room (5°C) in the lab for 24 h. Maximum quantum efficiency of PSII (Fv/Fm) was measured in the dark-adapted needles using a Pocket PEA (Hansatech Instruments, Norfolk, United Kingdom).

For the determination of the total N and C content and δ^13^C signature, needles were collected from one of the two cut branches per tree and five trees per site were used as replicates (*n* = 5). The needles were oven-dried (75°C, 5 days) and ground with a ball mill. An aliquot of approximately 2 mg of the fine powder produced per sample was used for the determination of total N and C content and δ^13^C composition according to Fotelli et al. (2003) using an isotope mass spectrometer (Delta plus, Finnigan MAT, Bremen, Germany) coupled to an elemental analyzer (NA1110, CE-Instruments, Rodano, Milan, Italy). Acetanilide (10.36% N and 71.09% C) was used as the standard (Merck KGaA, Darmstadt, Germany). The carbon isotope ratio (δ^13^C; ‰) of each sample was then determined as

(2) $$\delta^{13}C=\left[\left(Rsample/Rstandard\right)-1\right]\times 1000$$

where *R*_*sample*_ is the ^13^C/^12^C ratio of the sample and *R*_*standard*_ is the ^13^C/^12^C ratio of the Vienna Pee Dee Belemnite (VPDB) standard.

### Determination of the Infestation Level

At the infested site (Sani), one additional branch per tree was collected using a telescopic scissor to assess the level of infestation by *M. hellenica.* Each branch was put in a separate plastic bag and stored in a cold room (5°C) until measurements. The stored samples were examined macroscopically and stereoscopically when needed. The number of nymphs was counted and the size (length and diameter) of each branch was measured to calculate its bark area. Finally, the number of nymphs was expressed on a bark surface area (cm^2^) basis, according to Speight (1991).

### Metabolic Analyses

Based on the seasonal variation in *M. hellenica* number of nymphs (Figure 2), metabolic profiling of the needles of infested and non-infested trees was focused on the period when the number of nymphs increased (April to June) to assess metabolic changes during the peak of their feeding activity. In addition, needles collected in March and July were analyzed to characterize the periods preceding and following the maximum feeding activity of the nymphs. Needle samples of five trees per treatment (control vs. infested) were used for metabolic profiling. Needles were collected from one of the two branches used for the determination of physiological parameters, frozen in liquid nitrogen, and stored at −80°C until further analysis.

Metabolites of needles were extracted and derivatized using a modification of the method described by Kreuzwieser et al. (2009). Approximately 50 mg of homogenized frozen plant material was suspended with 300 μl of methanol (100%). Thereafter, 30 μl of ribitol (2 mg ml^–1^) was added as internal standard, samples were incubated at 70°C for 15 min and shaken at 1400 r.p.m. Two hundred microliters of chloroform was added and samples were incubated for 5 min at 37°C and 1400 r.p.m. and, subsequently, centrifuged for 5 min and 15,000 *g*. An aliquot of 80 μl of the supernatant was lyophilized overnight. For derivatization, 20 μl of a 20 mg ml^–1^ solution of methoxyamine hydrochloride in anhydrous pyridine (Sigma-Aldrich Inc., Steinheim, Germany) was added to the dried extracts. Samples were incubated for 90 min at 30°C and 1400 *g* before 35 μl of N-methyl-N-(trimethylsilyl)-trifluoroacetamide (MSTFA; Sigma-Aldrich) was added and samples were derivatized at 37°C for 30 min and with shaking at 1400 r.p.m.

Derivatized samples of needle extracts were analyzed with a G.C.–M.S. system (Agilent GC 6890N coupled to a 5975C quadrupole MS detector; Agilent Technologies, Palo Alto, CA, United States), equipped with an autosampler (MultiPurpose Sampler MPS2; Gerstel, Mülheim, Germany) and controlled by the Agilent MASSHUNTER software (Agilent Technologies). For this purpose, 1-μl aliquots were injected in splitless mode into the system and metabolites were separated on a capillary column (HP-5 ms ultra-inert, 0.25 mm ID, 0.25 lm film thickness, 30 m length; Agilent Technologies). Run conditions as well as MS settings were as described by Kreuzwieser et al. (2009). For peak identification and detection of compounds, the Golm Metabolome Database was used in combination with the Quantitative Analyses software (Agilent Technologies). Peak areas of plant material were normalized using the peak area of Ribitol and the fresh weight of samples. Common contaminants and artifact peaks were identified by blanks and were subtracted from sample peaks.

### Statistical Analysis

Two branches per tree were used for the physiological measurements and one branch per tree was used for determining the infestation level. In the former case, a mean of the values obtained by the two branches per tree was calculated and the individual trees were used as replicates (for *A*_*max*_, *g*_*s*_, WUE_*i*_, RWC: *n* = 6 or 7, for the control and the infested site, respectively). For the metabolic analyses and the needle δ^13^C composition and total N, C content, five trees were used as replicates for both sites (*n* = 5) and samples were collected from one branch per tree.

Statistical analysis was performed using R (R Core Team, 2019). Significant differences between treatments (control vs. infested trees) at each time point (month) were assessed with Welch two-sample *t* test. The relationships between parameters were assessed with linear and non-linear regression analyses. To identify significant differences in infestation intensity between months, one-way ANOVA and Tukey’s test were performed. Changes in the abundances of selected metabolites were visualized with the pheatmap package in R (Kolde, 2019).

## Results

### Climatic Conditions

**FIGURE 1:**
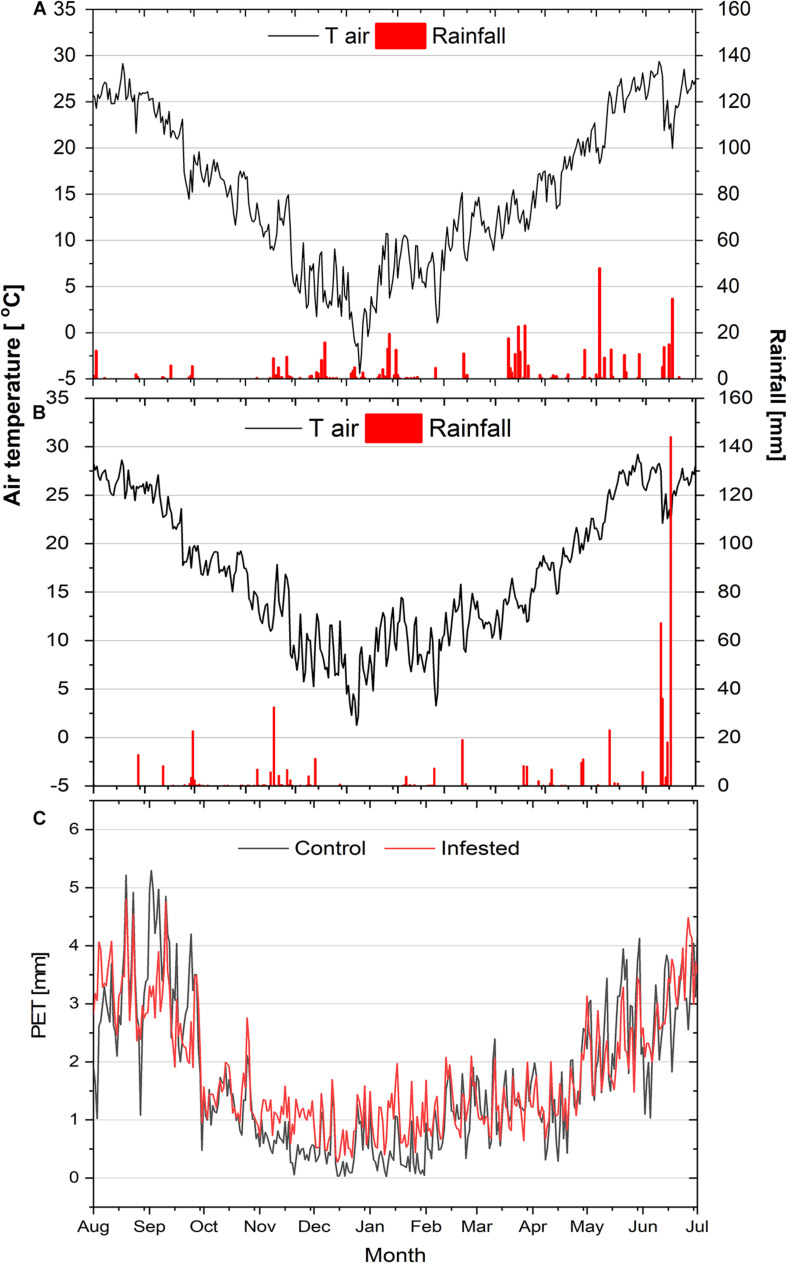
Seasonal courses of mean daily air temperature (*T*_*air*_) and daily precipitation (rainfall) at the control site **(A)** and at the infested site **(B)**, and of mean daily potential evapotranspiration (PET) at the two sites **(C)**.

The ombrothermic diagrams of the study sites are depicted in Supplementary Figure 1 and the seasonal courses of air temperature, rainfall and potential evapotranspiration during the study year in Figure 1. Air temperature presented similar seasonal variation among the two sites, except for the minimum winter temperature (January 2019), which was lower at the control, compared to the infested site. Likewise, total rainfall did not differ noticeably between the two study sites, amounting to 464 mm and 512 mm at the control and infested site, respectively.

However, a difference between the sites was observed for the summer rainfall that was almost equally distributed in June and July at the control site, but took place almost exclusively in July at the infested site. Finally, mean daily potential evapotranspiration (PET) during the study period at the two sites, calculated according to Allen et al. (1998), was also comparable (1.83 vs. 1.90 mm day^–1^ for the control and the infested site, respectively).

### Level of Infestation

**FIGURE 2:**
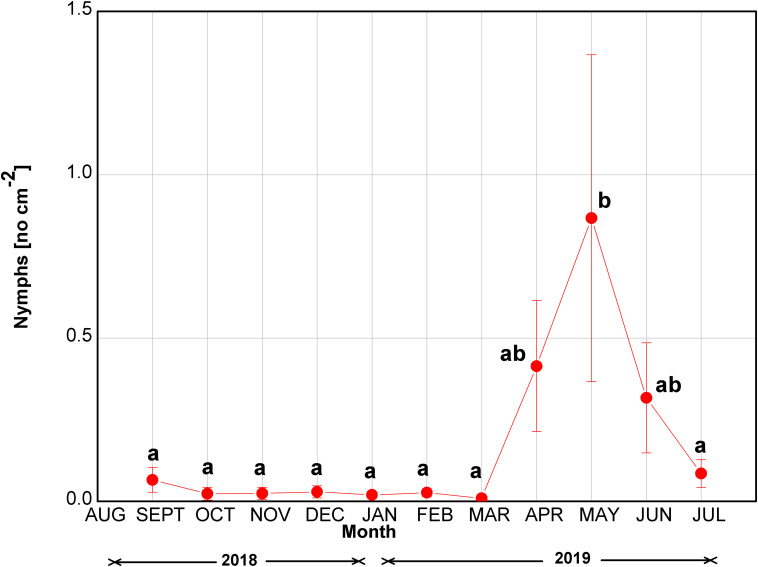
Seasonal course of the number of nymphs of *M. hellenica* at the infested site. Symbols represent mean values ± SE (*n* = 7). Different letters indicate statistically significant differences among months (*p* < 0.05).

The number of *M. hellenica* nymphs was constantly close to zero until March 2019 (Figure 2). Nymph numbers increased in April at the start of parthenogenesis, peaked in May, and gradually diminished until the end of the investigation in July. Although the increase in the nymphs’ abundance was substantial during April–June 2019, the difference was statistically significant only in May, when the insect’s number of nymphs peaked.

### Gas Exchange and Water Balance

**FIGURE 3:**
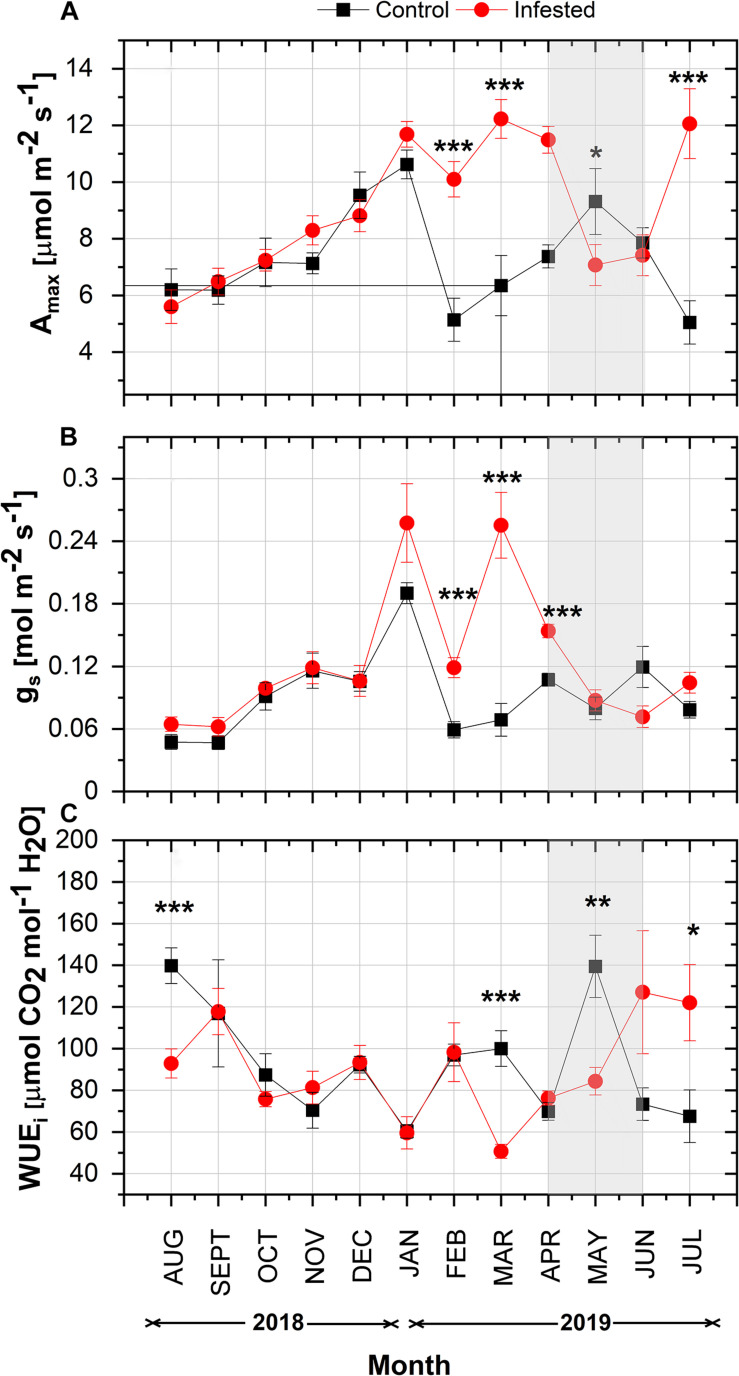
Seasonal course of **(A)** *A*_*max*_, **(B)** *g*_*s*_, and **(C)** WUE_*i*_ in pine needles at the control and the infested site. Symbols represent monthly means ± SE (*n* = 6 and 7 for the control and the infested site, respectively). The light gray shaded area indicates the intense feeding period of *M. hellenica* (April–June), according to Figure 2. Statistically significant differences between the treatments are indicated by (*) for *p* < 0.05, (**) for *p* < 0.01, and (***) for *p* < 0.001.

In both study areas, maximum net photosynthetic assimilation (*A*_*max*_) showed a similar seasonal increase from August 2018 to January 2019 (Figure 3A). From February 2019 onward, *A*_*max*_ was significantly different between the two sites. *A*_*max*_ of infested pines peaked in March and was higher than that of healthy pines until April 2019, when the intense feeding activity of nymphs started. Thereafter, *A*_*max*_ of infested trees declined substantially and was lower than the controls until June 2019. *A*_*max*_ of infested trees peaked again when the number of nymphs was almost zero in July. At the control site, *A*_*max*_ followed a more typical seasonal fluctuation for Aleppo pine; it decreased in mid-winter (February), then peaked in May, and declined during summer months.

In line with *A*_*max*_, *g*_*s*_ followed the same increasing pattern from August until January with no difference among treatments (Figure 3B). Then, from February until April, *g*_*s*_ was significantly higher in infested than in control trees and declined substantially during the months of high nymph abundance. A slight recovery of *g*_*s*_ was observed after the number of nymphs fell to zero.

Intrinsic water use efficiency, i.e., the ratio of *A*_*max*_ to *g*_*s*_, showed similar seasonal variation between the study sites until February 2019 (Figure 3C). Following the changes in *A*_*max*_ and *g*_*s*_, the pattern of WUE_*i*_ differed between infested and non-infested trees from March 2019 onward. It is noteworthy that WUE_*i*_ of infested trees gradually increased from March until the end of the intense infestation period, when the abundance of nymphs was high.

Maximum quantum yield of PSII (Fv/Fm) exhibited small seasonal fluctuations and no substantial differences between needles of infested and healthy Aleppo pines (Supplementary Figure 3). Fv/Fm was also maintained at high values (above 0.8) in both treatments almost throughout the study period.

At both study sites, a significant relationship was found between *g*_*s*_ and *A*_*max*_ (*R*_*adj*_^2^ = 0.55 and *R*_*adj*_^2^ = 0.61, *p* < 0.05 at the control and the infested site, respectively; Supplementary Figure 4), pointing to a close stomatal control on *A*_*max*_. Moreover, the substantial decline of both *A*_*max*_ and *g*_*s*_ in the infested trees was closely related to the concurrent variation in the number of *M. hellenica* nymphs, as indicated by the significant negative effect of nymph abundance on both *A*_*max*_ and *g*_*s*_ (Supplementary Figures 5A,B).

**FIGURE 4:**
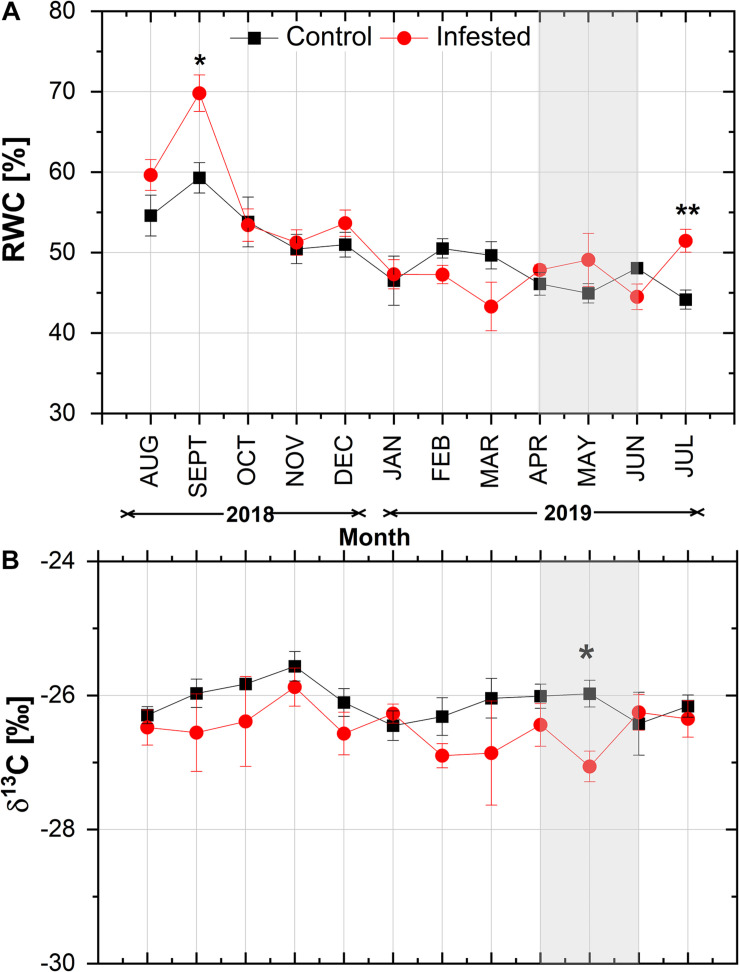
Seasonal course of **(A)** relative water content (RWC) and **(B)** δ^13^C in pine needles at the control and the infested site. Symbols represent monthly means ± SE (RWC: *n* = 6 and 7 for the control and the infested site, respectively; δ^13^C: *n* = 5). The light gray shaded area indicates the intense feeding period of *M. hellenica* (April–June), according to Figure 2. Statistically significant differences between the treatments are indicated by (*) for *p* < 0.05 and (**) for *p* < 0.01.

With the exception of September and July, needle RWC did not differ significantly between infested and healthy trees (Figure 4A). RWC exhibited a similar declining pattern from August to January in both sites, followed by a slight seasonal fluctuation. During the peak in *M. hellenica* nymph abundance (April–June), opposite responses of RWC were observed between control and infested trees. Similarly, with the exception of May, needle δ^13^C did not differ significantly between infested and healthy trees (Figure 4B) and exhibited a comparable seasonal variation at both sites.

**FIGURE 5:**
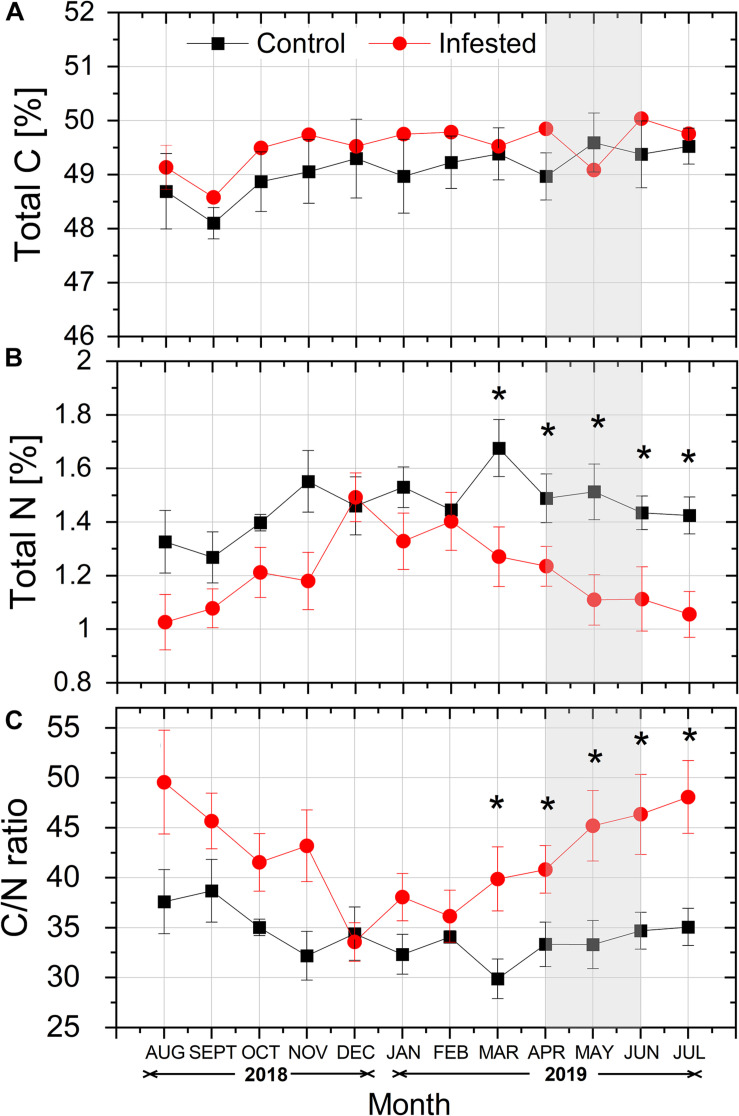
Seasonal course of **(A)** total C content (%), **(B)** total N content (%), and **(C)** C/N ratio in pine needles at the control and the infested site. Symbols represent monthly means ± SE (*n* = 5). The light gray shaded area indicates the intense feeding period of *M. hellenica* (April–June), according to Figure 2. Statistically significant differences between the treatments are indicated by (*) for *p* < 0.05.

Total needle C content (%) tended to be higher in infested vs. healthy trees throughout the study period, except from May 2019, but the differences among treatments were not significant (Figure 5A). On the contrary, total N content (%) was lower in the needles of infested vs. healthy pines, and this difference was significant from March to July, during the intense feeding activity of *M. hellenica* nymphs (Figure 5B). Finally, the differences in needles’ total C and mostly total N contents led to significantly enhanced C/N ratios in infested trees, compared to the controls, during the same period (Figure 5C).

### Metabolic Changes

**FIGURE 6:**
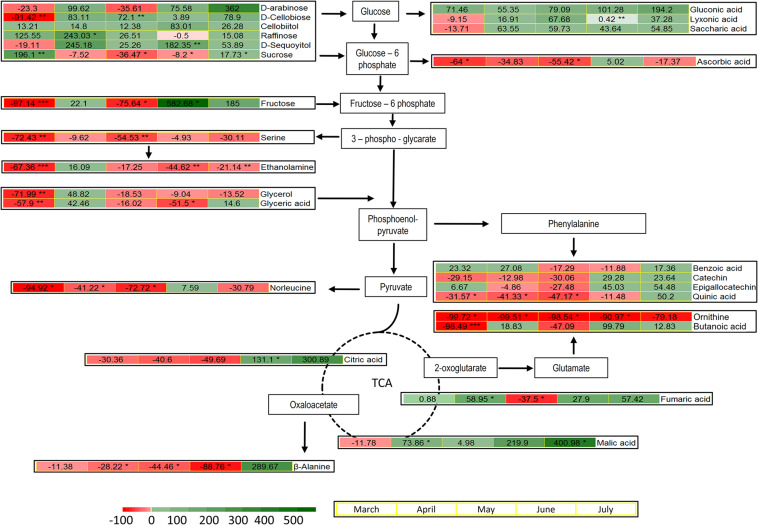
Pathways and heatmaps of selected metabolites presenting % changes in infested trees, relative to the controls, during the intense feeding period of *M. hellenica* nymphs (April–June), prior to this period (March), and in the recovery phase (July). Accumulation of metabolites is indicated with green scale color, while depletion is indicated with red scale color. Statistically significant induced changes are indicated by (*) for *p* < 0.05, (**) for *p* < 0.01, and (***) for *p* < 0.001.

The metabolites identified by means of GC-MS included 11 organic acids, with three metabolites of the tricarboxylic acid (TCA) cycle, 12 sugars and derivatives, six amino acids, four phenolic compounds, and phosphoric acid (Supplementary Table 1). The induced changes in the abundance of metabolites due to infestation by *M. hellenica*, compared to healthy Aleppo pine trees, were tested at five different time points (March, April, May, June, and July), based on the observed changes in the number of the insect’s nymphs (Figure 2) and the concurrent responses of gas exchange parameters (Figure 3). April, May, and June corresponded to the nymphs’ maximum feeding activity, while March and July were used as periods preceding and following the maximum feeding activity of the nymphs. The infestation-induced changes in the abundance of all identified metabolites at each time point, compared to the control, were expressed as percentages (%) of the respective control (Table 2). In addition, the changes in the abundances of selected metabolites are depicted in Figure 6 in relation to metabolic pathways.

**TABLE 2:** Changes^(1)^ (%) in the abundance of all identified metabolites in needles of infested Aleppo pines, relative to the controls, during the period March–July.

	March	April	May	June	July
**ORGANIC ACIDS**					
***Tricarboxylic acids***					
Citric acid	–30.36	–40.60	–49.69	131.10*	300.89
Fumaric acid	0.88	58.95*	−37.50*	27.90	57.42
Malic acid	–11.78	73.86*	4.98	219.90	400.98
***Sugar acids***					
Ascorbic acid	−64.00*	–34.83	−55.42*	5.02	–17.37
Galactonic acid	–34.68	–17.87	3.89	33.04*	–19.99
Gluconic acid	71.46	55.35	79.09	101.28	194.20
Glyceric acid	−57.90**	42.46	–16.02	−51.50*	14.60
Lactobionic acid	–28.87	22.85	–2.14	−48.38**	–16.80
Lyxonic acid	–9.15	16.91	67.68	0.42**	37.28
Monomethyl ester phosphoric acid	–39.49	10.28	–29.74	–64.91	–24.44
Saccharic acid	–13.71	63.55	59.73	43.64	54.85
OTHER ACIDS					
Phosphoric acid	−51.86*	57.58	–13.20	–1.59	–30.15
AMINO ACIDS					
β-Alanine	–11.38	−28.22*	−44.46*	8−8.76*	289.67
4-Amino-butanoic acid	−98.49***	18.83	–47.09	99.79	12.83
Ethanolamine	−67.36***	16.09	–17.25	−44.62**	−21.14**
Norleucine	−94.92*	−41.22*	−72.72*	7.59	–30.79
1,5-Lactam-ornithine	−99.72*	−99.51*	−98.54*	−90.97*	–79.18
Serine	−72.43**	–9.62	−54.53**	–4.93	–30.11
**SUGARS**					
Cellobiitol	13.21	14.80	12.38	83.01	26.28
D-Cellobiose	−91.42**	83.11	72.10**	3.89	78.90
Fructose	−97.14***	22.10	−75.64*	582.68*	185.00
D-β-Galactopyranosyl-1,3 arabinose	–23.30	99.62	–35.61	75.58	362.00
Galactose	−98.66***	–32.30	14.98	14.04	–5.97
Gentiobiose	–63.40	–34.94	–55.51	–10.30	–14.26
Glycerol	−71.99**	48.82	–18.53	–9.04	–13.52
Maltose	−50.84*	−35.91*	−58.44**	29.95	12.21
Myo-Inositol	–16.76	10.52	36.31	–14.55	–9.30
D-Pinitol	11.59	–17.48	–15.49	–15.13	27.15
Raffinose	125.55	243.03*	26.51	–0.50	15.08
D-Sequoyitol	–19.11	245.18**	25.26	182.35	53.89
Sucrose	196.10**	–7.52	−36.47*	−8.20*	17.73*
**PHENOLICS**					
Benzoic acid	23.32	27.08	–17.29	–11.88	17.36
Catechin	–29.15	–12.98	–30.06	29.28	23.64
Epigallocatechin	6.67	–4.86	–27.48	45.03	54.48
Quinic acid	−31.57*	−41.33*	−47.17*	–11.48	50.20

*^1^Statistically significant induced changes are indicated with bold numbers and with (^∗^) for p < 0.05, (^∗∗^) for p < 0.01, and (^∗∗∗^) for p < 0.001.*

A pronounced change was observed in the level of sucrose, which followed the same pattern as *A*_*max*_. It decreased when the number of *M. hellenica* nymphs peaked and increased before and after this peak in March and June (Supplementary Figure 6). Other carbohydrates were also elevated during the high infestation period (arabinose, cellobiitol, D-cellobiose, fructose, raffinose, D-sequoyitol) or showed both increasing and decreasing responses (myo-inositol, D-pinitol, maltose, galactose, glycerol). On the other hand, gentobiose was reduced in infested trees relative to control throughout the study period. Likewise, several organic acids (saccharic, gluconic, and lyxonic acid) were up-regulated throughout the study period, while others were both up- and down-regulated (glyceric, lactobionic).

Moreover, TCA cycle intermediates, such as malic and fumaric acid, were augmented during most of the months investigated, while citric acid was increased in June and July.

The levels of all identified amino acids declined due to infestation during most of the studied months. The only noticeable increase observed was the level of β-alanine at the recovery phase in July.

Phenolic compounds were induced in infested trees toward the end of the high infestation period (catechin, epigallocatechin) and during recovery (benzoic acid, quinic acid).

## Discussion

The present study investigated the responses of Aleppo pine to the infestation by the honeydew-producing insect *M. hellenica*. The impacts of the insect’s attack range from pine growth declines and partial canopy desiccation to individual tree necroses, when combined with other abiotic and biotic stress factors (Yeşil et al., 2005; Gallis, 2007; Mendel et al., 2016). These negative effects are recorded in eastern Mediterranean countries, where the giant pine scale is naturally distributed and particularly in Greece, where it was artificially introduced for promoting pine honey production. In regions recently invaded by the insect such as Australia (Avtzis et al., 2020) and Croatia (Masten Milek et al., 2019), where its natural enemies are absent, the impacts on Aleppo pine as well as other host conifers can be exacerbated. The expected intense drought events at xerothermic environments where *P. halepensis* is either native or planted may limit the species’ potential for successful defense responses against the infestation by *M. hellenica*. However, limited information is available on the physiological and metabolic responses of Aleppo pine to the infestation by this pine scale to date. For this purpose, we assessed gas exchange and water status in combination with metabolic profiling and identification of changes of metabolite abundance in needles of Aleppo pines attacked by the giant pine scale during an entire year.

### Seasonal Regulation and Limitation of Photosynthesis in the Absence of Drought

In healthy Aleppo pines, *A*_*max*_ peaked in May, as previously recorded in eastern Mediterranean regions (Klein et al., 2013; Fotelli et al., 2019). On the contrary, infested Aleppo pines limited their photosynthetic rates during the intense feeding period of the giant scale (April–June; Figure 2), but maximized *A*_*max*_ quite early in the growing season (March) and in mid-summer (July) (Figure 3A). Both healthy and infested pines also maintained high *A*_*max*_ rates during winter months (December and January), in line with the species’ ability to fully exploit warm and sunny winter days in terms of assimilation (Sperlich et al., 2015; Klein et al., 2016; Fotelli et al., 2019). Indeed, during the measuring days of these months, *T*_*air*_ ranged from 10.4 to 14.3°C. Aleppo pine acclimates its optimum temperature of photosynthesis to the growth temperature as long as no temperature extremes occur (Sperlich et al., 2019). Moreover, the species overcomes water shortage and capitalizes mild winter days by stomatal regulation to minimize water loss and by low sensitivity to photoinhibition (Martínez-Ferri et al., 2000; Sperlich et al., 2014). The decline in *A*_*max*_ of healthy trees in February could be due to the absence of rainfall during the preceding 3-week period. On the contrary, under the same climatic conditions, *A*_*max*_ of infested trees decreased only slightly. The maintenance of high Fv/Fm values (Supplementary Figure 3) and close stomatal control (Supplementary Figure 4) in infested pines seems to enable seasonal optimization of *A*_*max*_ and support Aleppo pine’s high plasticity not only to abiotic, but also to biotic stresses, such as the infestation by *M. hellenica*. The infested pines maximized their photosynthetic rates prior to the intense feeding activity of the emerging nymphs, and recovered high *A*_*max*_ again in July taking advantage of both the reduced pressure by the insect’s nymphs (Figure 2) and the rainfalls of this period, despite the simultaneous high temperatures (Figure 1). Thus, one of the main responses of Aleppo pine to the giant pine scale attack seems to be the re-arrangement of the seasonal variation of its photosynthetic activity.

The limited photosynthetic rates during the insect’s intense feeding and the negative effect of the insect’s abundance on *A*_*max*_ (Supplementary Figure 5) contradict the argumentation of Crawley (1999) and Retuerto et al. (2004) that attack by sap-sucking scale insects leads to increased host photosynthesis to compensate for the losses in carbohydrates. In line with our results, other plant–insect interactions resulted in reduced gas exchange of the host plant (Gretchen et al., 1992; Bueno et al., 2009). Reduced photosynthesis has been generally associated with plant defense responses to biotic stresses (Bilgin et al., 2010; Göhre et al., 2012) and is considered to be a plant-directed adaptive response. This view is supported by studies on the down-regulation of genes encoding enzymes of carbon fixation (Kerchev et al., 2012). It is, thus, important to elucidate the mechanism leading to the reduction of *A*_*max*_ during the intense infestation period. Limitation of *A*_*max*_ was probably mediated by partial stomatal closure (Figures 3A,B), as supported by the close relationship between *A*_*max*_ and *g*_*s*_ (Supplementary Figure 4B). Petrakis et al. (2010) suggested that the attack by *M. hellenica* may lead to structural modifications in the tracheids, which eventually could hinder water transport and lead to desiccation – as observed by other insects (Mendel and Liphschitz, 1988). We, however, detected no drought stress caused by infestation, as shown by the absence of differences in RWC and natural δ^13^C signature between infested and healthy Aleppo pines (Figure 4). The same was supported by the maintenance of Fv/Fm values of infested Aleppo pine needles above 0.80 (Supplementary Figure 3; e.g., Taïbi et al., 2017; Banks, 2018).

Therefore, our first hypothesis that infestation by *M. hellenica* results in impaired gas exchange in Aleppo pine is partly verified. However, we also observed compensatory responses in Aleppo pine, achieved by adjustments in its seasonal fluctuation of *A*_*max*_. In addition, although related to stomatal closure, the limited assimilation during the intense infestation of Aleppo pines was not due to desiccation. Partitioning of carbon resources to needles may explain the decline in photosynthesis as explained in the next section.

### Metabolic Defense Is Coordinated by the Accumulation of Sugars

Sucrose, the main end product of photosynthesis, follows the same seasonal pattern as *A*_*max*_ in the needles of infested Aleppo pines (Supplementary Figure 6). This response may also be due to sucrose transport from the needles to the sinks created by the phloem feeding activity of the nymphs and could explain the reduction of needle total C content at the peak of infestation in May (Figure 5A). On the other hand, many other carbohydrates and sugar acids (such as raffinose, D-sequoyitol, arabinose, D-cellobiose, cellobiitol, lyxonic, gluconic, and saccharic acid) were induced in the needles of infested Aleppo pines upon the outbreak of infestation in April and/or later (fructose, arabinose) during the main feeding period of the nymphs (Figure 6). Given that *M. hellenica* mainly feeds on the phloem sap of the trunk and branches (Gounari, 2006), partitioning of carbohydrates to Aleppo pine needles may facilitate tolerance to the infestation. Kerchev et al. (2012) similarly reported that allocation of carbon resources to structures unavailable to insects may be linked to plant tolerance to insect herbivory.

The mechanism that explains the high carbohydrate induction in the needles on infested Aleppo pines may be similar to that imposed by environmental stresses. Drought resulted in reduced mobilization of carbon in the leaves (Sala et al., 2010). Drought and exposure to ozone were also associated with impaired sugar loading and transport in phloem to sites of demand (Lemoine et al., 2013; Hesse et al., 2019) and, consequently, with accumulation of carbohydrates in the leaves/needles of broadleaf and conifer trees, leading to inhibition of photosynthesis (Granda and Camarero, 2017; Piper et al., 2017; Chen et al., 2018). Similarly, exposure to biotic stress caused accumulation of carbohydrates in the leaves of the host plant, a response proposed to signal reduced photosynthesis (Chou et al., 2000). Accumulation of sugars in the needles and reduced photosynthesis could also result in increased internal CO_2_ concentration and mediate stomatal closure (e.g., Kottapalli et al., 2018), as supported by the simultaneous decrease in stomatal conductance, accumulation of sugars, and maintenance of higher total C in the needles of infested vs. healthy trees during the attack period (Figures 3B, 5A, 6).

The high concentration of the three carbohydrates raffinose, D-cellobiose, and D-sequoyitol underlines the idea that the plant’s defense mechanisms were activated because they play important signaling roles in plant innate immunity (Valluru and Van den Ende, 2011; Bolouri Moghaddam and Van den Ende, 2012; Souza et al., 2017). The induction of carbohydrates in response to biotic stresses, the so-called “high sugar plant resistance,” may fuel glycolysis and the tricarboxylic acid (TCA) cycle for the production of energy and secondary metabolites, needed in plant defense (Morkunas and Ratajczak, 2014).

The level of tricarboxylic acids, like malic, fumaric, and citric acid, were indeed augmented during different phases of *M. hellenica* nymph accumulation (Figures 2, 6). These TCA intermediates are able to prime plant defense against pathogens (Balmer et al., 2018) and are also found to accumulate in response to leaf herbivory by caterpillars (Papazian et al., 2019). Moreover, the TCA cycle is fundamental for the provision of energy and carbon for the synthesis of amino acids and phenolic compounds essential in plant defense.

However, most amino acids identified in our study were down-regulated during the high infestation period (April–June; Table 2 and Figure 6), as similarly observed by Papazian et al. (2019) in response to insect herbivory. The depletion in amino acids could be partially attributed to the infestation-induced stomatal closure, which may have resulted in impaired N supply to the foliage, as indicated by the declining needle N content of attacked pines during the intense infestation period (Figure 5B). Reduced transpiration, by abiotic stresses like drought, is known to result in limited N uptake and N transport to the tree’s canopy (Gessler et al., 2017). An additional explanation for the depletion of amino acids, despite the enhanced TCA cycle, is their utilization for the production of other metabolites, such as phenolics produced from phenylalanine. Indeed, catechin, epigallocatechin, benzoic acid, and quinic acid were accumulated in infested vs. healthy needles toward the end of the intense nymph feeding activity (June) or even during the recovery phase (July) (Figure 6). These phenolic compounds are involved in plant defense and were augmented in poplar leaves and in the phloem of Aleppo pine in response to pathogens (Wang et al., 2016; Ullah et al., 2017; Morcillo et al., 2019), while they are also shown to be toxic to herbivores (Barbehenn and Constabel, 2011). Moreover, high catechin abundance in different organs of Aleppo pine has been associated with enhanced antimicrobial and antibacterial defense (Salim et al., 2019).

Ascorbic acid was depleted in needles of infested vs. healthy trees, almost throughout the infestation period (Table 2 and Figures 2, 6). Enhancement of ascorbic acid usually occurs during stomatal closure, under, e.g., drought, to scavenge the reactive oxygen species (ROS) produced with low intercellular CO_2_ concentration (Rennenberg et al., 2006). However, the defense-induced limited stomatal conductance in our study was not followed by such a response, consistent with Arab et al. (2016) who reported reduced ascorbic acid concentrations in the xeromorphic date palm in response to drought and heat. Nevertheless, the induction of phenolics at the end of the insect’s feeding period may counteract the depletion of ascorbic acid and improve the antioxidative capacity of the host pines.

In summary, our second hypothesis that infestation by the giant pine scale induces metabolic disorders in Aleppo pine is rejected. Although ascorbic acid and amino acids were depleted, other metabolic changes included the accumulation of carbohydrates, TCAs, and phenolics, which can be seen as a successful defense of Aleppo pine against the insect’s herbivory.

## Conclusion

This is a first record on the ecophysiological and metabolic responses of Aleppo pine against the attack by the sap-sucking insect *M. hellenica*. Aleppo pine exhibited a cascade of defense mechanisms. The core included the limitation of photosynthesis during the peak of infestation and its compensatory stimulation in periods preceding and following the insect’s high feeding activity, under otherwise suboptimal environmental conditions. Defense reactions of both primary and secondary metabolism were observed. Accumulation of carbohydrates in the needles of infested trees signaled stomatal closure and photosynthetic inhibition, while induction of TCA intermediates enabled the production of phenolics, necessary in defense against biotic stress. The employment of defense responses by Aleppo pine during intense attack by the pine scale and recovery potential at its end may enable re-establishment of physiological functions and long-term resilience. Such a defense strategy may explain why the long-term occurrence of *M. hellenica* in eastern Mediterranean countries may have negative impacts on Aleppo pine, but does not seem to threaten its existence under the current climate. Future research should focus on diverse Aleppo pine forests and pine species to elucidate whether these responses are site- or species-specific and if they ensure defense of host pines in regions of the world recently invaded by *M. hellenica*, as well as under a drier and warmer climate.

## Data Availability Statement

The raw data supporting the conclusions of this article will be made available by the authors, without undue reservation.

## Author Contributions

MF and KR conceived this research. MF and DA designed this research. MF, FL, DA, and DM collected the data. MF, FL, GS, DM, and AP carried out the data analysis. MF, FL, and GS contributed to the data visualization. MF, HR, AP, and KR provided resources. MF wrote the manuscript. HR, AP, and DA reviewed the manuscript. All authors contributed to the article and approved the submitted version.

## Conflict of Interest

The authors declare that the research was conducted in the absence of any commercial or financial relationships that could be construed as a potential conflict of interest.
