# Supplementary material for: Effective Defense of Aleppo Pine Against the Giant Scale Marchalina hellenica Through Ecophysiological and Metabolic Changes
Source: Front Plant Sci. 2020 Dec 10;11:581693. doi: 10.3389/fpls.2020.581693 (PMC7758410; doi:10.3389/fpls.2020.581693)
Supplement: Supplementary file 1 [file Data_Sheet_1.pdf]

Supplementary Table S1. Metabolites identified from Aleppo pine needles.

ORGANIC ACIDS	Mass Factor	Retention time	Quantification ion (m/z)
Tricarboxylic acids			
Citric acid (4TMS)	73.6	27.40	273.1
Foumaric acid (2TMS)	71.4	17.51	245.1
Malic acid (3TMS)	70.2	20.83	73.0
Sugar acids			
Ascorbic acid (4TMS)	72.5	29.48	374.2
Galactonic acid (6TMS)	79.1	30.36	73.0
Gluconic acid (6TMS)	78.8	30.64	73.0
Glyceric acid (3TMS)	74.1	17.32	292.1
Lactobionic acid (TMS) BP	71.0	37.12	201.1
Lyxonic acid (5TMS)	75.1	26.18	333.1
Monomethylester phosphoric acid (2TMS)	76.2	13.40	241.1
Saccharic acid (6TMS)	71.9	31.08	333.1
OTHER ACIDS			
Phosphoric acid (3TMS)	90.6	15.94	299.1
AMINO ACIDS			
β-Alanine (3TMS)	83.9	19.35	247.6
4-Amino-butanoic acid (3TMS)	82.9	21.54	173.6
Ethanolamine (3TMS)	79.7	15.58	173.6
Norleucine (2TMS)	83.1	15.78	158,0
1,5 lactam-Ornithine	86.3	20.02	243.1
Serine (3TMS)	85.0	18.00	218.1
SUGARS & DERIVATIVES			
Cellobiitol (9TMS)	77.0	39.70	244.1
D-Cellobiose (8TMS)	83.4	45.05	204.1
Fructose (1MEOX) (5TMS) MP	87.3	28.45	217.1
D – β – Galactopyranosyl-1,3 Arabinose (1MEOX) (7TMS)	70.2	40.15	204.1
Galactose (1MEOX) (5TMS) BP	80.8	29.19	204.6
Gentobiose (1MEOX) (8TMS) BP	70.8	43.25	204.1
Glycerol (3TMS)	70.7	15.95	73.0
Maltose (1MEOX) (8TMS) BP	73.2	43.39	360.7
Myo-inositol (6TMS)	84.3	31.77	217.1
D-Pinitol (5TMS)	89.4	27.69	259.6
Raffinose (11TMS)	86.0	47.82	360.7
D-Sequoyitol (5TMS)	84.4	29.77	217.1
Sucrose (8TMS)	88.1	39.48	217.1
PHENOLICS			
Benzoic acid (1TMS)	75.0	14.93	179.0
Catechin (5TMS)	83.6	42.03	369.2
Epigallocatechin (6TMS)	87.6	42.53	456.2
Quinic acid (5TMS)	77.4	41.08	344.7

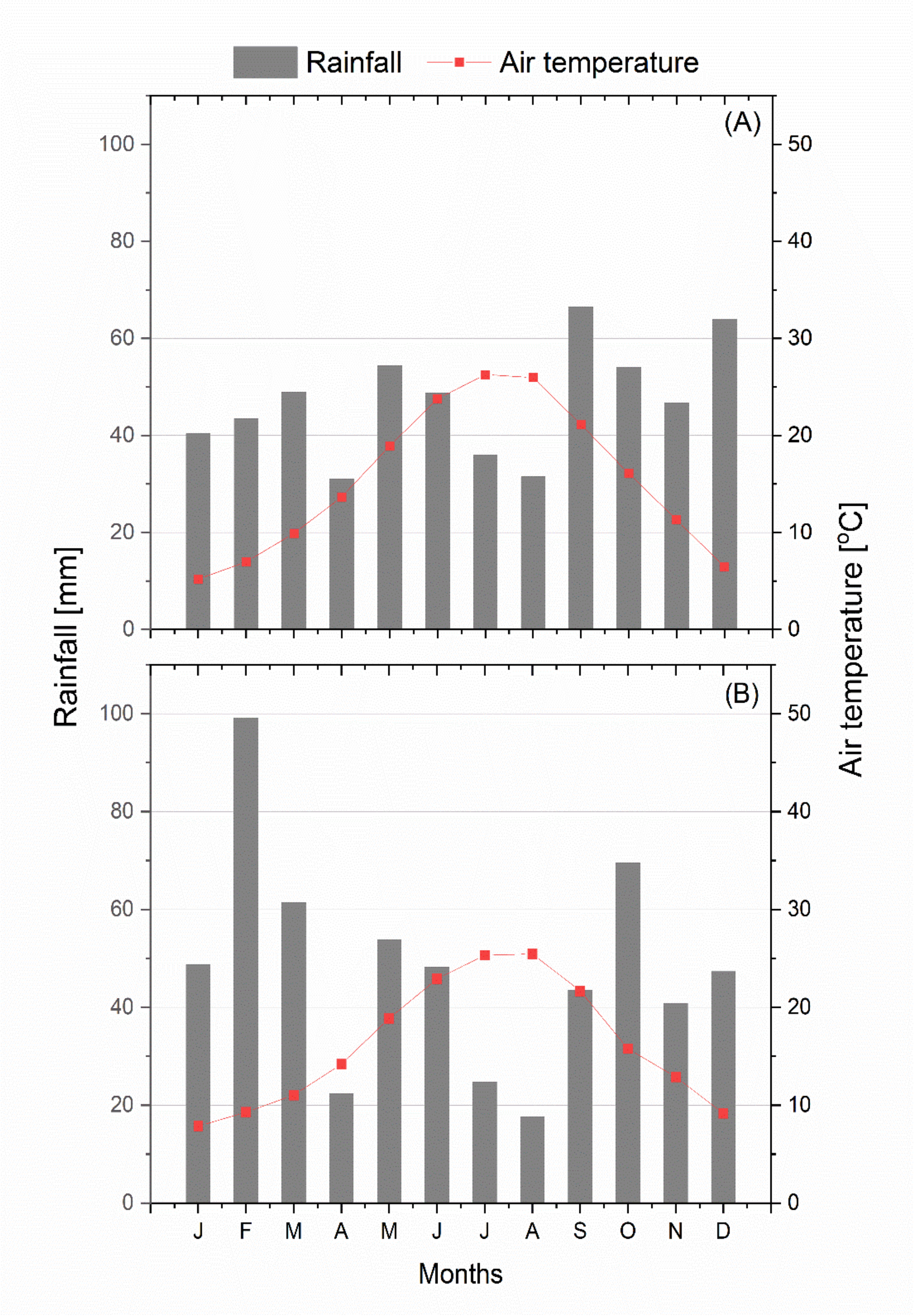

Supplementary Figure S1. Ombrothermic diagrams of (A) the control Aleppo pine site at FRI and (B) the infested Aleppo pine site at Sani. Mean monthly air temperature and monthly rainfall are averages of the periods 2002-2018 and 2009-2018 for the control and the infested site, respectively.

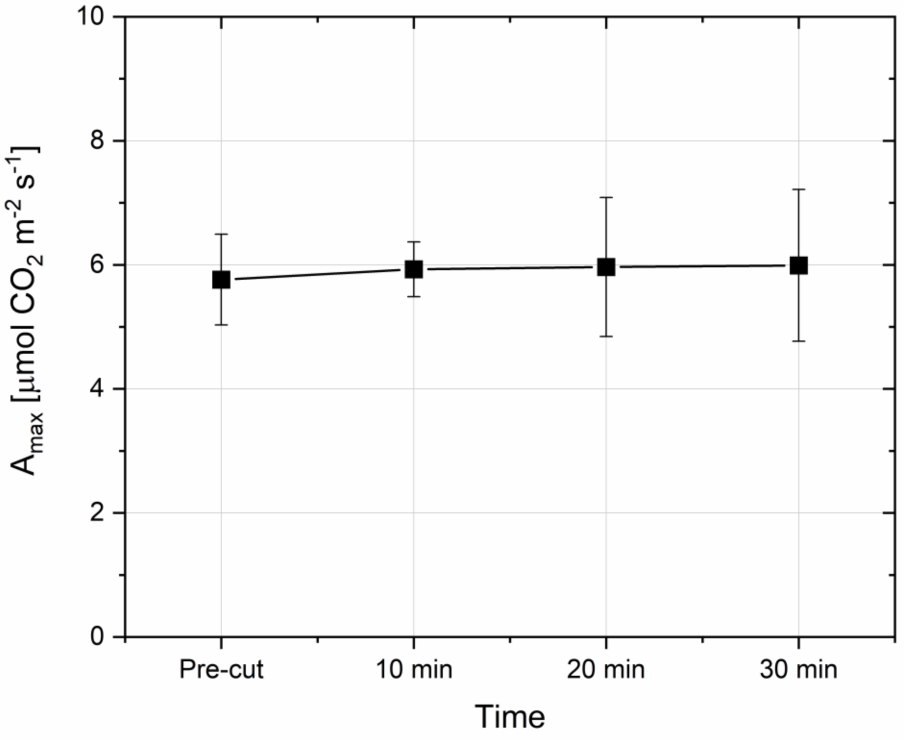

Supplementary Figure S2. A_{max} measured before and after branch abscission in low canopy sunlit branches of Aleppo pine trees at the control site (FRI). Symbols represent mean values ± SE of three trees.

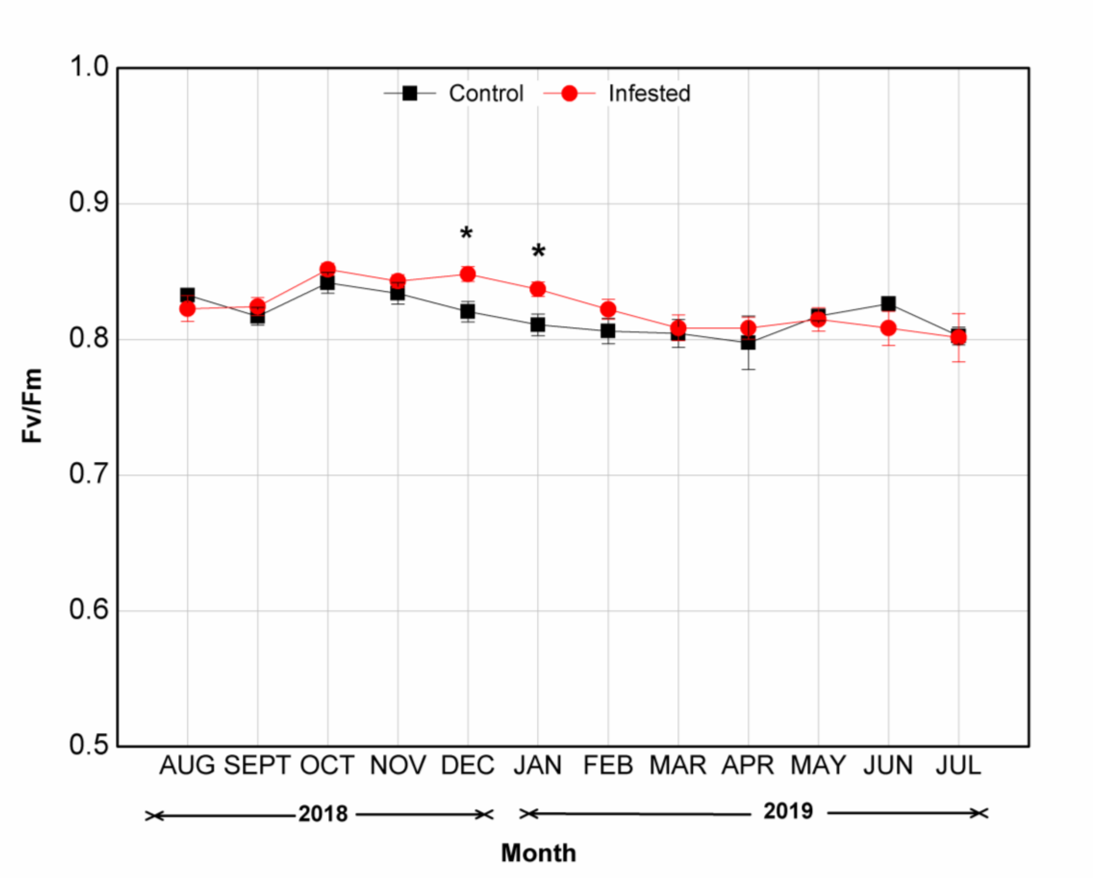

Supplementary Figure S3. Seasonal course of Aleppo pine needles' maximum quantum efficiency of PSII (Fv/Fm) at the control and the infested sites. Symbols represent monthly means ± SE of six and seven trees for the control and infested site, respectively. Statistically significant differences are indicated by (*) for $p < 0.05$.

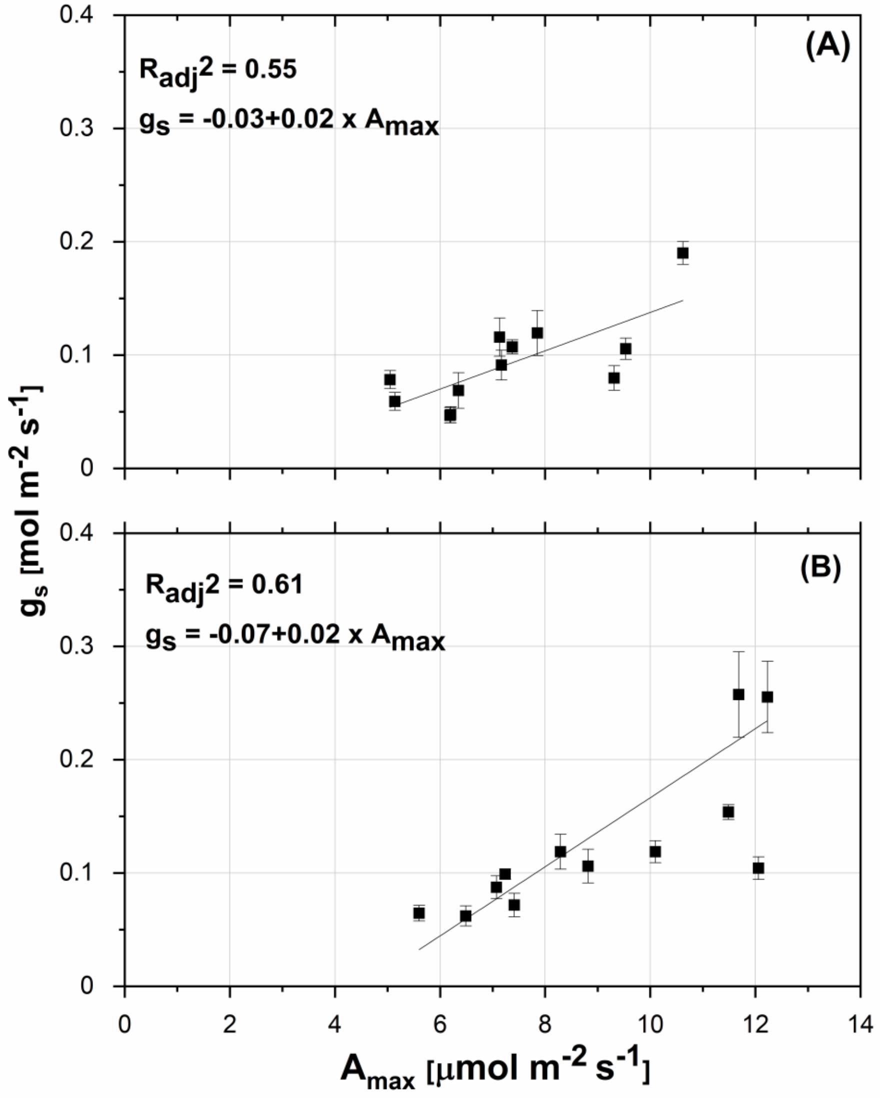

Supplementary Figure S4. Regression models describing the significant relationships ($p<0.05$) between stomatal conductance (g_s) and maximum photosynthesis (A_{max}) of Aleppo pine needles at the control site (A) and the infested site (B).

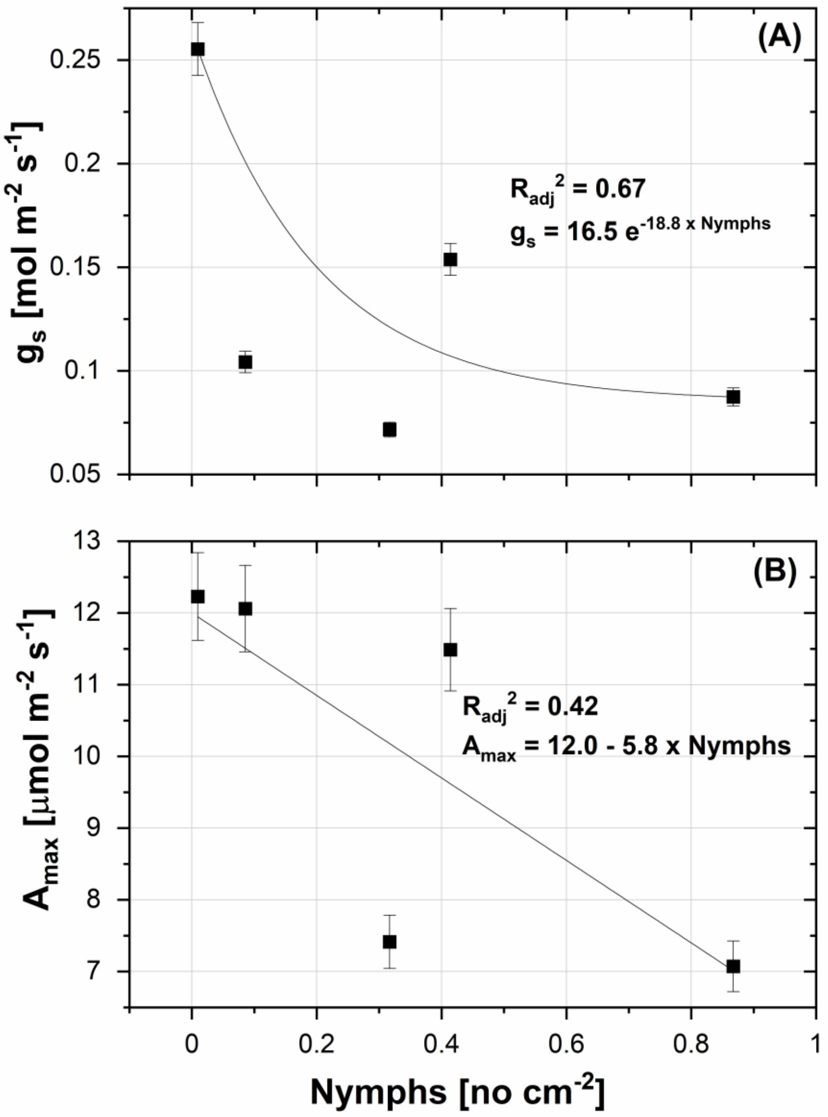

Supplementary Figure S5. Regression models describing the significant relationships ($p<0.05$) (a) between maximum photosynthesis (A_{max}) and the number of *M. hellenica* nymphs, (b) stomatal conductance (g_s) and the number of *M. hellenica* nymphs during the period March – July 2019.

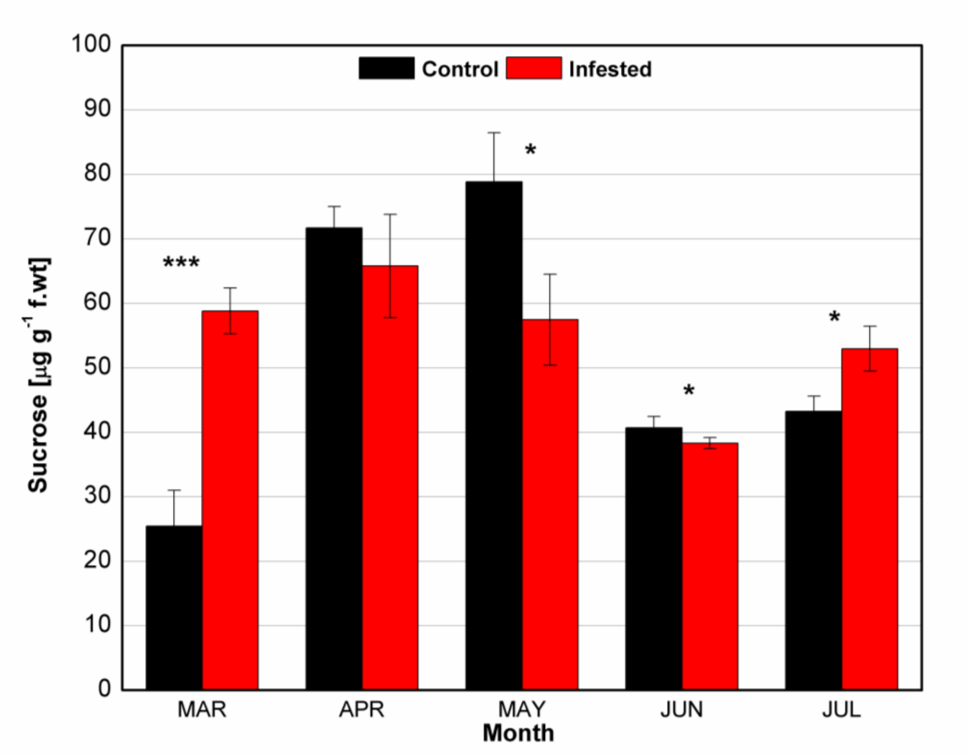

Supplementary Figure S6. Sucrose concentration (µg g^{-1}f.wt) in the needles of control and infested Aleppo pine trees. Bars represent means ± SE (n=5). Statistically significant differences between the treatments are indicated by (*) for p< 0.05 and (***) for p<0.001
